# Simulated virtual reality experiences for predicting early treatment response in panic disorder

**DOI:** 10.3389/fdgth.2025.1684001

**Published:** 2025-11-06

**Authors:** Byung-Hoon Kim, Jae-Jin Kim, Junhyung Kim, Jiook Cha, Sang-Won Jeon, Kang-Seob Oh, Dong-Won Shin, Sung Joon Cho

**Affiliations:** 1 Department of Psychiatry, Yonsei University College of Medicine, Seoul, Republic of Korea; 2 Institute of Behavioral Sciences in Medicine, Yonsei University College of Medicine, Seoul, Republic of Korea; 3 Department of Psychiatry, Kangbuk Samsung Hospital, Sungkyunkwan University School of Medicine, Seoul, Republic of Korea; 4 Workplace Mental Health Institute, Kangbuk Samsung Hospital, Sungkyunkwan University School of Medicine, Seoul, Republic of Korea; 5 Department of Brain and Cognitive Sciences, Seoul National University, Seoul, Republic of Korea; 6 Interdisciplinary Program in Artificial Intelligence, Seoul National University, Seoul, Republic of Korea; 7 Department of Psychology, Seoul National University, Seoul, Republic of Korea; 8 Institute of Psychological Science, Seoul National University, Seoul, Republic of Korea

**Keywords:** virtual reality, panic disorder, early treatment response, machine learning, anxiety, heart rate variability, VR-based assessments

## Abstract

**Background:**

Panic disorder (PD) is a disabling anxiety condition in which early improvement during treatment can predict better long-term outcomes.

**Objectives:**

This study investigated whether a newly developed virtual reality-based assessment tool, the Virtual Reality Assessment of Panic Disorder (VRA-PD), can help predict early treatment response in individuals with PD.

**Methods:**

In total, 52 participants, including 25 patients diagnosed with PD and 27 healthy individuals, were evaluated every 2 months over a 6-month period. Assessments included self-reported anxiety levels and heart rate variability measured during virtual reality scenarios, as well as standard clinical questionnaires. Patients with PD were further categorized based on their treatment progress into early responders (*n* = 7) and delayed responders (*n* = 18). A machine-learning model (CatBoost) was used to classify participants into early responder, delayed responder, and healthy control groups.

**Results:**

The model that combined virtual reality-based and conventional clinical data achieved higher accuracy (85%) and F1-score (0.71) than models using only clinical (accuracy: 77%, F1-score: 0.56) or only virtual reality data (accuracy: 75%, F1-score: 0.64). The most important predictors included anxiety levels during virtual scenarios, heart rate variability metrics, and scores from clinical scales such as the Panic Disorder Severity Scale and Anxiety Sensitivity Index.

**Conclusions:**

This study highlights the value of virtual reality-based assessments for predicting early treatment outcomes in PD. By providing ecologically valid and individualized measures, virtual reality may enhance clinical decision-making and support personalized mental healthcare.

## 1 Introduction

Virtual reality (VR) is an emerging technology with the potential to enhance and innovate diagnostic and therapeutic evaluations of mental health (1). Its immersive and controlled environment recreates clinical scenarios where patients can exhibit natural responses in a safe and regulated space (2). VR has been effectively applied in exposure-based therapies for anxiety disorders (3) and post-traumatic stress disorder (4), demonstrating that simulated VR experiences closely replicate real-world clinical practices (5). Moreover, VR facilitates the collection of behavioral and physiological data, such as heart rate variability (HRV), enabling reliable and ecologically valid assessments of patient responses (6).

Panic disorder (PD), classified as an anxiety disorder, is characterized by unexpected panic attacks—sudden episodes of intense anxiety accompanied by various physiological and cognitive symptoms without identifiable external triggers. This condition causes significant distress and functional impairment (7). Affecting approximately 2%–3% of the population, PD imposes a substantial burden on individuals and healthcare systems (8, 9). Early treatment response (ETR) in pharmacotherapy for PD is critical because failure to achieve early response may delay the onset of therapeutic benefit. Such delays can prolong the acute phase, increasing the risk of adverse events, premature discontinuation, or insufficient effectiveness (10–12). Identifying ETR allows for timely treatment adjustments, potentially improving outcomes and minimizing unnecessary exposure to ineffective regimens. Monitoring ETR enables clinicians to proactively adjust or introduce antidepressants during the acute phase, reducing the risk of relapse or recurrence (13). Thus, predicting ETR is pivotal for tailoring treatment strategies and improving patient outcomes.

The potential to utilize VR to develop assessments with high ecological validity and safety offers new opportunities to predict ETR and address the limitations of traditional assessments in clinical practice for PD. Conventional methods, such as questionnaires and clinician-based ratings or interviews, have shown limited success in predicting treatment response (14, 15). In contrast, integrating biological and behavioral data is increasingly recognized as essential for understanding and predicting treatment outcomes in mental disorders (16). This approach has demonstrated promise in related areas; for instance, ecological data, such as neuroimaging and molecular markers, significantly improved predictions of antidepressant treatment outcomes in depression compared to conventional models (17). In the context of PD, VR is particularly effective in objectively evaluating the transient nature of panic attacks, avoidance behaviors, and associated physiological responses. Unlike conventional self-report methods, which rely on subjective recall and lack ecological validity, VR-based assessments capture real-time behavioral and physiological metrics (18). As such, VR-based ecological assessments may contribute significantly to the prediction of ETR in PD.

This study investigated whether a VR-based tool can predict treatment response at 2 months, correlating with ETR in patients with PD. The Virtual Reality Assessment of Panic Disorder (VRA-PD), validated in distinguishing patients with PD from healthy controls (HCs) through behavioral and biological markers in anxiety-inducing and relaxation scenarios (18), is utilized to develop a model that classifies patients with PD as early or delayed responders and identifies HCs. We hypothesized that behavioral metrics and HRV collected through the VRA-PD will effectively predict treatment response within 2 months.

## 2 Materials and methods

### 2.1 Sample and setting

Participants were enrolled between September 2021 and January 2023, with follow-up conducted every 2 months for 6 months from September 2021 to July 2023. The study included 52 individuals: 24 patients diagnosed with PD and 28 HCs. Patients with PD were recruited from outpatient clinics at the Department of Psychiatry, Korea University Guro Hospital. Psychiatric evaluations were conducted using the Mini-International Neuropsychiatric Interview 5.0 (MINI 5.0) to assess for psychiatric conditions and substance use disorders (19). The study prioritized recruiting patients with PD undergoing treatment rather than drug-naïve, first-episode patients, reflecting real-world clinical settings where treatment response can be monitored over 2 months. Efforts were made to exclude comorbid depression due to its significant impact on treatment outcomes and prognosis in PD (20, 21).

Eligibility for the PD group required: (i) a diagnosis of PD based on the MINI structured interview; (ii) age between 19 and 50 years; and (iii) voluntary participation with written informed consent. Exclusion criteria included: (i) neurological disorders, other psychiatric illnesses, significant physical health conditions, or serious infections; (ii) alcohol or substance abuse/dependence; (iii) current or past diagnoses of major depressive episodes, bipolar I disorder, or psychotic disorders; (iv) pregnancy; and (v) difficulty using VR equipment.

For the HC group, inclusion criteria were: (i) age between 19 and 50 years; (ii) absence of psychiatric symptoms or medication use; and (iii) voluntary participation with signed informed consent. Exclusion criteria were: (i) neurological or psychiatric disorders, major physical illnesses, or severe infections; (ii) alcohol or substance abuse/dependence; (iii) history of neurological or psychiatric disorders; (iv) pregnancy; and (v) difficulty using VR equipment. HCs were recruited through online and print advertising and carefully screened to ensure adherence to the concept of “healthy” (i.e., meeting no exclusion criteria). The HC group was matched with the PD group by age and sex to ensure comparability.

A 6-month longitudinal study design was used to assess treatment outcomes and predict treatment response at 2 months. The study included initial and follow-up in-depth interviews, clinical assessments, and VR-based assessments using the VRA-PD. Over the 6-month period, VR-based and conventional assessments, excluding the baseline MINI, were conducted identically every 2 months. At baseline, participants completed sociodemographic questionnaires and provided clinical history details, including the duration of PD and pharmacotherapy components (Table 1). All patients received routine care, including consultations for prescriptions, but they did not participate in psychotherapy sessions, such as cognitive behavioral therapy or similar therapeutic interventions. To account for antidepressants affecting PD treatment outcomes, standardized daily antidepressant doses were recorded by converting them into fluoxetine-equivalent doses following the methodology of Hayasaka et al. (22). For example, 40 mg/d of fluoxetine corresponded to 34.0 mg/d of paroxetine and 18.0 mg/d of escitalopram.

**Table 1:** Schedule of clinical and virtual reality (VR)-based assessments across study timepoints.

Timepoint	Clinical interviews	Clinical scales	VR-based assessment
Baseline	MINI PDSS	LSAS, GAD-7, HADS, IUS, ASI, BFNE, PSWQ	VRA-PD modules (AS + HRV)
2 months	PDSS	LSAS, GAD-7, HADS, IUS, ASI, BFNE, PSWQ	VRA-PD modules (AS + HRV)
4 months	PDSS	LSAS, GAD-7, HADS, IUS, ASI, BFNE, PSWQ	VRA-PD modules (AS + HRV)
6 months	PDSS	LSAS, GAD-7, HADS, IUS, ASI, BFNE, PSWQ	VRA-PD modules (AS + HRV)

MINI, mini-international neuropsychiatric interview 5.0; PDSS, panic disorder severity scale; LSAS, liebowitz social anxiety scale; GAD-7, generalized anxiety disorder scale; HADS, hospital anxiety and depression scale; IUS, intolerance of uncertainty scale; ASI, anxiety sensitivity index; BFNE, brief fear of negative evaluation; PSWQ, Penn State worry questionnaire; VRA-PD, virtual reality assessment of panic disorder; AS, subjective anxiety scores; HRV, heart rate variability.

All procedures were conducted in compliance with the local ethics committee's approval at Korea University Guro Hospital (approval number 2021-GR0057; approval date July 12, 2021). Participants provided written informed consent after being thoroughly informed about the study's aims, procedures, and potential risks. The study adhered to the principles of the Declaration of Helsinki (1964) for ethical research conduct. Furthermore, to ensure participant confidentiality, all data were anonymized, access was restricted to authorized researchers, and all sensitive information was encrypted and securely stored in compliance with relevant data protection regulations.

### 2.2 Measures

#### 2.2.1 Outcomes

The primary outcome, ETR, was operationally defined as a reduction of 40% or more in Panic Disorder Severity Scale (PDSS) scores at 2 months from baseline, based on structured clinical interviews and PDSS assessments (23–26). Patients were categorized into early-response (ER) and delayed-response (DR) groups accordingly, with HCs included for comparison.

The primary analytic aim of this study was to evaluate whether integrating VR-based behavioral and physiological data with conventional clinical measures improves the prediction of ETR. Secondary outcomes included between-group comparisons of anxiety and heart rate variability metrics, as well as SHapley Additive exPlanations (SHAP)-based feature importance analyses to identify key predictors.

#### 2.2.2 Predictors

We utilized variables from two key domains as predictors: the conventional domain and the VR-based domain measured concurrently using the VRA-PD.

The conventional domain included established clinical scales, such as the Liebowitz Social Anxiety Scale (LSAS) (27, 28), Generalized Anxiety Disorder Scale (GAD-7) (29, 30), Hospital Anxiety and Depression Scale (HADS) (31, 32), Intolerance of Uncertainty Scale (IUS) (33, 34), Anxiety Sensitivity Index (ASI) (35, 36), Brief Fear of Negative Evaluation (BFNE) (37, 38), and Penn State Worry Questionnaire (PSWQ) (39, 40), all administered at baseline. These instruments are widely validated measures of anxiety and provide a comprehensive profile of participants' anxiety symptoms. Sociodemographic variables, such as age, sex, marital status, years of education, average alcohol consumption (drinks per day), and smoking habits (packs per day), were also included.

The VR-based domain involved subjective anxiety scores (AS) and HRV features collected through the VRA-PD. The VRA-PD has been validated as a reliable tool for assessing anxiety-related behaviors in patients with PD (18). The system configuration, operation, and content of each VRA-PD module were identical to those described in the validation study (18). The configuration diagram of the VRA-PD is shown in Supplementary Figure S1, and screenshots with descriptions of each module are presented in the Supplementary Figure S2 and Supplementary Text.

VRA-PD comprises four modules: “Baseline evaluation (M0)”, “Daily environment exposure (M1)”, “Relaxation (M2)”, and “Interoceptive exposure (M3)”. Through the VRA-PD, participants input AS, a subjective measure of their anxiety experience, while HRV data are measured at specific intervals. Participants rate their AS on a visual analog scale (VAS) ranging from “not at all” (0 points) to “very much” (100 points). HRV data are collected using a photoplethysmogram sensor [Model: ubpulse H3 (Pulse Analyzer, MFDS, Certification No. 11-1296), LAXTHA Inc., Daejeon, South Korea] placed on the index finger. Five key HRV parameters included well-known metrics associated with anxiety, such as the ratio of low-frequency to high-frequency power (LF/HF), very low-frequency power (VLF), root mean square of successive differences (RMSSD), standard deviation of NN intervals (SDNN), and total power (TP) (41–43). A total of 50 VR-based variables (10 AS and 40 HRV features) were used as predictors (Supplementary Table S1).

### 2.3 Analyses

#### 2.3.1 Statistical analysis

Group comparisons of demographic and clinical characteristics were conducted using non-parametric tests due to the small sample size of the ER group (*n* = 7). The Kruskal–Wallis *H*-test was employed to examine differences across the three groups (HC, ER, and DR), followed by Mann–Whitney *U*-tests with Bonferroni correction for pairwise *post-hoc* comparisons. Although non-parametric tests were used, descriptive statistics are presented as means and standard deviations for consistency with previous literature and to facilitate comparisons across studies.

For the evaluation of machine-learning model performance, we employed a three-class classification approach, where the model was trained to distinguish between HC, ER, and DR. Performance metrics (precision, recall, and F1-score) were calculated for each group separately as well as for overall model performance. For the HC group, these metrics represent the model's ability to distinguish non-patients from patients with PD. Specifically, precision for the HC group indicates the proportion of instances predicted as HC that were correctly classified, while recall represents the proportion of actual HC participants correctly identified by the model.

The receiver operating characteristic (ROC) curves were generated by extracting binary classification performance from our multi-class models (specifically for ER vs. non-ER classification) to evaluate the discriminative power of the different predictive domains. Area under the curve (AUC) values were calculated to quantify model performance, with higher values indicating better discriminative ability.

#### 2.3.2 Machine-learning model training

We trained a machine-learning classification model to predict the three treatment response groups—HC, ER, and DR—using predictors obtained from the initial assessment. No dropouts occurred between baseline and the 2-month follow-up period, ensuring the completeness of data for this phase of the study. Specifically, we trained a CatBoost classifier model, an advanced tree-based model (44). CatBoost was selected for its ability to handle categorical features natively and its robustness in mitigating overfitting, particularly in studies with small and imbalanced sample sizes (44, 45). As an ensemble learning method based on gradient boosting over decision trees, CatBoost constructs a forest of trees to enhance predictive performance by capturing complex non-linear relationships and interactions within the data (46).

To address the limited sample size, we used leave-one-out cross-validation (LOOCV), which is particularly suitable for small datasets because it maximizes the use of available information and reduces the risk of overfitting (47). Classification performance was quantified using precision and the F1 score. The F1-score, which balances precision and recall, provided a robust measure of performance, particularly for classes with imbalanced samples. Final precision and F1 scores were averaged over all LOOCV iterations to comprehensively evaluate the model's ability to classify each group accurately. Confusion matrices were plotted to visually assess true positives, false positives, true negatives, and false negatives for each class. Model training, testing, and visualization were implemented using the scikit-learn (v0.24.1) (48) and catboost (v1.2.7) (44) Python libraries.

#### 2.3.3 Input feature importance evaluation

The contribution of each input feature to prediction was assessed using SHAP feature importance approach, applied to the aggregated findings to evaluate the relevance of predictors for the group classification task (49). SHAP is a model-agnostic technique for analyzing feature significance, employing a game-theoretic approach. By calculating SHAP values, the contribution of each input feature to the final model prediction was quantified, with feature significance determined as the average of the absolute SHAP values across aggregated samples.

## 3 Results

### 3.1 Participant demographics and psychometric scale scores

The demographic and clinical characteristics of each group are summarized in Table 2. Among demographic variables, only years of education showed a significant difference across groups (H = 9.37, *P* = 0.009). In terms of anxiety-related measures, all scales except for the LSAS revealed significant differences between groups. *post-hoc* analysis indicated that compared to the HC group, the ER and DR groups exhibited significantly higher symptoms of PD as measured by the PDSS. Specifically, the DR group scored significantly higher than the HC group on all anxiety-related measures except for the LSAS. In contrast, the ER group differed significantly from the HC group only on the GAD-7, the anxiety subscale of the HADS, and the ASI. No significant differences were observed between the ER and DR groups on any anxiety-related measures, except for years of education, where the ER group had relatively higher educational levels.

**Table 2:** Group differences in demographics and anxiety-related clinical characteristics.

Variable	HC (*n* = 27)		ER (*n* = 7)		DR (*n* = 18)		H-value^†^	post-hoc test^‡^		
	Mean	SD	Mean	SD	Mean	SD	*P*-value	HC vs. ER	HC vs. DR	ER vs. DR
Age (years)	33.3	9.61	39.71	8.06	33.33	11.5	2.52 (.283)	.414	1.000	.406
Education (years)	15.15	1.61	15.43	1.9	13.11	2.37	9.37 (.009)	1.000	.016	.021
Smoking (pack/d)	0.09	0.27	0.3	0.37	0.33	0.48	7.00 (.030)	.130	.080	1.000
Alcohol (unit/wk)	4.97	7.16	7.97	6.38	5.61	6.21	2.19 (.334)	.417	1.000	.841
PDSS	0	0	16.14	4.22	14.11	3.18	44.98 (<.001)	<.001	<.001	1.000
LSAS-fear	21.26	15	21	14.09	31.5	15.24	5.12 (.077)	1.000	.087	.455
LSAS-avoidance	19.74	13.07	21.14	17.1	30.06	15.99	4.460 (.108)	1.000	.118	.610
GAD	3.67	3.61	10	5.6	12.28	5.51	22.76 (<.001)	.007	<.001	1.000
HADS-anxiety	4.63	2.98	11.43	3.41	13.28	5.03	28.22 (<.001)	<.001	<.001	.862
HADS-depression	6.93	3.97	9.71	4.57	11.94	3.65	14.37 (<.001)	.480	<.001	.644
IUS	27.19	7.96	30.14	8.97	36.11	6.74	11.73 (.003)	1.000	.002	.217
ASI	11.41	9.43	34.57	13.24	39.44	13.47	29.43 (<.001)	.004	<.001	1.000
BFNE	32.07	7.3	34.14	5.21	38	5.37	8.67 (.013)	1.000	.010	.580
PSWQ	46.33	14.01	59.43	16.4	65.22	11.71	16.23 (<.001)	.129	<.001	1.000

HC, healthy control; ER, early response; DR, delayed response; PDSS, panic disorder severity scale; LSAS, liebowitz social anxiety scale; GAD-7, generalized anxiety disorder scale; HADS, hospital anxiety and depression scale; IUS, intolerance of uncertainty scale; ASI, anxiety sensitivity index; BFNE, brief fear of negative evaluation; PSWQ, Penn State worry questionnaire.

† Kruskal–Wallis *H*-test was used instead of analysis of variation due to small sample size in the ER group (*n* = 7).

‡ Post-hoc comparisons were performed using Mann–Whitney *U*-tests with Bonferroni correction for multiple comparisons.

*P* < .05, statistically significant.

### 3.2 Participant demographics and psychometric scale scores

The significant results of the three-group ANOVA obtained within the VRA-PD are presented in Table 3, while results for all VRA-PD variables are provided in Supplementary Table S2. Significant group differences were identified for all seven AS measures. *Post-hoc* analysis revealed that AS was significantly higher in the DR group than that in the ER group in M1-P-AS, M2-P-AS, M1-E6-AS, M2-RR-AS, M3-P-AS, M3-HV-AS, and M3-DU-AS. The DR group also exhibited higher AS than the HC group across all virtual environments in all modules. However, no significant differences in AS were observed between the HC and ER groups.

**Table 3:** Significant group differences in anxiety scores and HRV parameters in VRA-PD.

Variable	HC (*n* = 27)		ER (*n* = 7)		DR (*n* = 18)		H-value^†^	post-hoc test^‡^		
	Mean	SD	Mean	SD	Mean	SD	*P*-value	HC vs. ER	HC vs. DR	ER vs. DR
M0-AS	10.74	20.37	4.29	5.35	33.33	33.95	8.04 (.018)	1.000	.029	.103
M1-P-AS	5.19	12.21	8.57	10.69	32.78	24.69	22.13 (<.001)	1.000	<.001	.059
M1-E2-AS	5.19	12.82	14.29	25.07	34.44	23.57	19.19 (<.001)	.672	<.001	.200
M1-E6-AS	5.93	12.79	17.14	24.98	46.67	29.31	22.91 (<.001)	.663	<.001	.106
M2-P-AS	4.81	12.21	5.71	7.87	40.00	27.65	24.25 (<.001)	1.000	<.001	.014
M2-RR-AS	4.07	10.47	4.29	7.87	30.56	27.33	20.50 (<.001)	1.000	<.001	.016
M2-MR-AS	2.22	8.01	2.86	4.88	21.67	23.07	19.20 (<.001)	1.000	<.001	.052
M3-P-AS	4.07	9.31	4.29	7.87	30.56	29.20	19.17 (<.001)	1.000	<.001	.018
M3-HV-AS	5.56	10.86	5.71	7.87	30.00	26.57	15.06 (<.001)	1.000	<.000	.075
M3-DU-AS	3.33	10.00	1.43	3.78	27.22	29.47	17.99 (<.001)	1.000	<.001	.010
M2-RR-SDNN	67.79	18.45	81.70	70.77	55.70	34.97	7.35 (.025)	1.000	.020	.641
M2-RR-VLF	864.47	923.28	1640.53	2599.15	411.88	424.18	7.27 (.026)	1.000	.048	.108
M3-HV-SDNN	58.71	30.10	56.36	19.54	42.27	26.53	7.15 (.028)	.211	.033	1.000

HC, healthy control; ER, early response group; DR, delayed response; VLF, very low frequency; SDNN, standard deviation of NN intervals.

† Kruskal–Wallis *H*-test was used instead of ANOVA due to small sample size in the ER group (*n* = 7).

‡ Post-hoc comparisons were performed using Mann–Whitney *U*-tests with Bonferroni correction for multiple comparisons.

*P* < .05, statistically significant.

Three HRV parameters showed significant differences across groups: M2-RR-SDNN, M2-RR-VLF, and M3-HV-SDNN. In particular, M2-RR-SDNN and M2-RR-VLF were significantly lower in the DR group compared to the HC group (*P* = 0.020 and 0.048, respectively), while no significant differences were observed between ER and DR groups after Bonferroni correction. Similarly, M3-HV-SDNN was lower in the DR group than in the HC group (*P* = 0.033), but not significantly different between ER and DR. These findings suggest that reductions in autonomic flexibility during VR-based interoceptive or relaxation tasks may be associated with delayed treatment response.

### 3.3 Performance of the prediction model by data domain

The performance of models utilizing the conventional, VR-based, and combined domains to predict early treatment response at 2 months in patients with PD is summarized in Table 4. The VR-based domain prediction model (precision = 0.75, macro-average F1 score = 0.64) demonstrated performance comparable to the conventional domain-based model (precision = 0.77, macro-average F1 score = 0.56). However, combining the two domains resulted in the highest performance, achieving the highest accuracy (precision = 0.85, macro-average F1 score = 0.71).

**Table 4:** Prediction model performance by data domain.

Dataset	Accuracy	Macro average F1 score	group	Precision	Recall	F1-score
Conventional domain	0.77	0.56	HC	1.00	1.00	1.00
			ER	0.00	0.00	0.00
			DR	0.65	0.72	0.68
VR-based domain	0.75	0.64	HC	0.77	0.89	0.83
			ER	0.33	0.29	0.31
			DR	0.87	0.72	0.79
Combined	0.85	0.71	HC	1.00	1.00	1.00
			ER	0.40	0.29	0.33
			DR	0.75	0.83	0.79

HC, healthy control; ER, early response; DR, delayed response.

For the conventional domain, the model achieved perfect precision, recall, and F1-score for the HC group but showed no predictive capability for the ER group. Prediction for the DR group was moderate, with a precision, recall, and F1 score of 0.65, 0.72, and 0.68, respectively. The VR-based domain model improved prediction performance for the DR group (precision = 0.87, recall = 0.72, F1 score = 0.79) and demonstrated modest improvements for the ER group, achieving an F1 score of 0.31. When combining both domains, the model maintained perfect prediction for the HC group, while showing improved precision (0.40), recall (0.29), and F1 score (0.33) for the ER group, along with comparable performance for the DR group (precision = 0.75, recall = 0.83, F1 score = 0.79).

**Figure 1:**
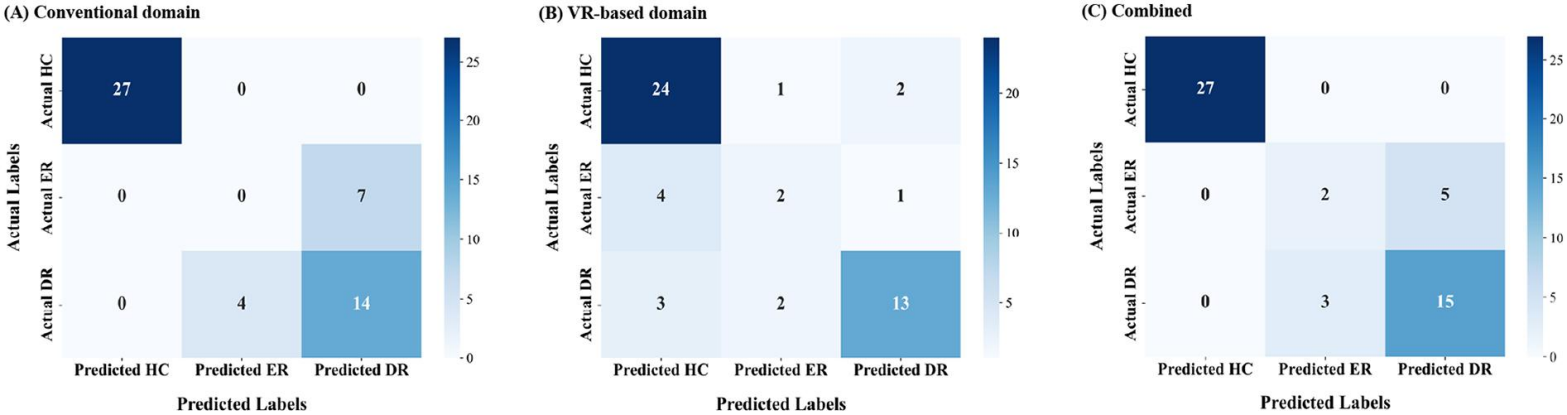
Confusion matrices for early treatment response (2 months) prediction across data domains. **(A)** Conventional domain. **(B)** VR-based domain. **(C)** Combined domain. VR, virtual reality.

Confusion matrices visualizing predictions relative to correct labels are shown in Figure 1 to illustrate classification errors. For the ER group, the conventional domain model misclassified all ER cases as DR (Figure 1A). In contrast, the VR-based model slightly improved performance, with some ER cases misclassified as HC instead of DR (Figure 1B). In the combined domain model, all misclassifications of ER cases were directed solely toward the DR group (Figure 1C). Despite these differences, the overall error rate for ER classification remained the same as in the VR-based model.

**Figure 2:**
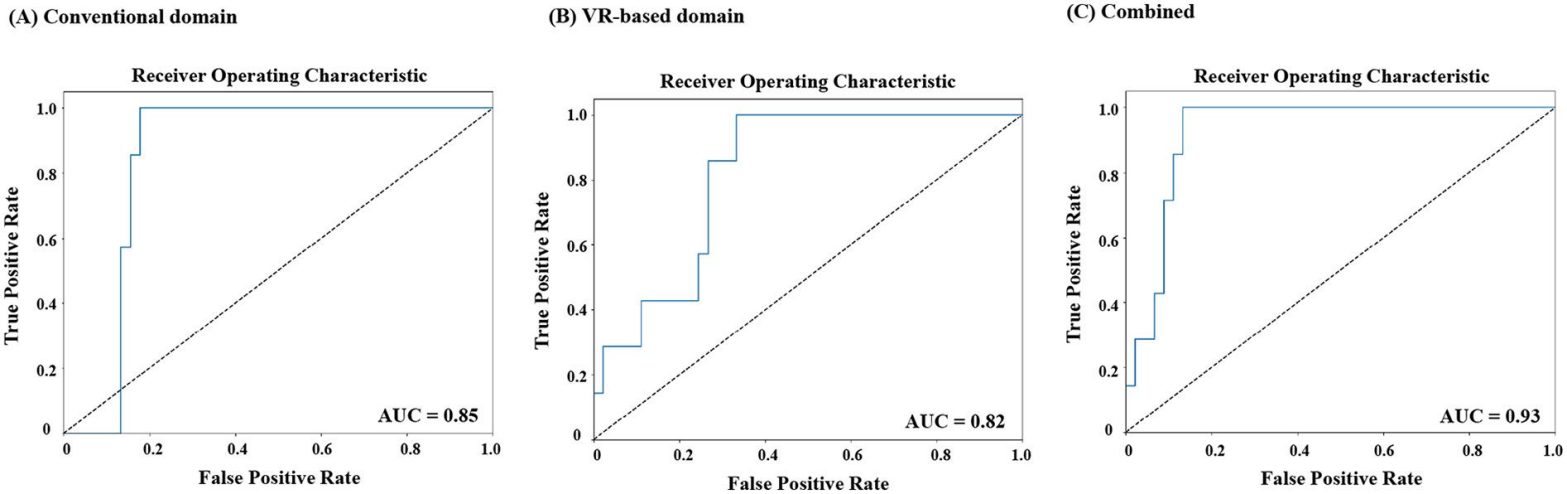
Receiver operating characteristic (ROC) curves for classification of early responders (ER) versus non-ER at 2 months across data domains. **(A)** Conventional domain. **(B)** VR-based domain. **(C)** Combined domain. ROC, receiver operating characteristic; ER, early response; VR, virtual reality.

**Figure 3:**
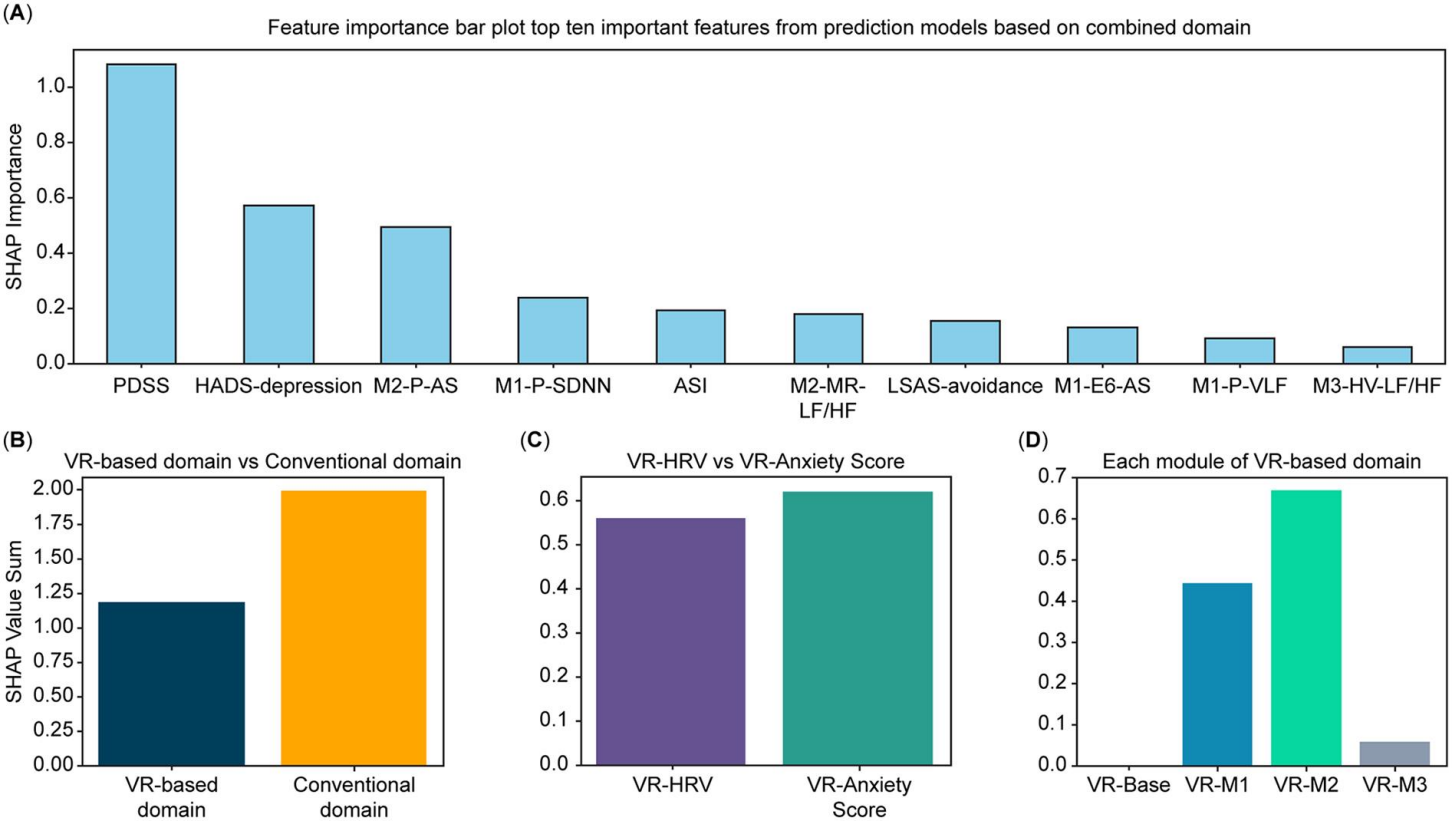
Feature-importance analysis derived from the CatBoost model. **(A)** Top 10 most important features selected from conventional and VR-based domains, ranked by SHAP values. **(B)** Comparative contribution of the conventional and VR-based domains to the total feature-importance sum. **(C)** Distribution of feature-importance sums across variable categories within the VR-based domain (subjective anxiety vs. HRV features). **(D)** Distribution of feature-importance sums across modules within the VR-based domain (Baseline [M0], Daily Environment Exposure [M1], Relaxation [M2], and Interoceptive Exposure [M3]). Feature-importance values were estimated using the SHAP method applied to the CatBoost model with leave-one-out cross-validation (LOOCV). VR, virtual reality; SHAP, SHapley Additive exPlanations; LOOCV, leave-one-out cross-validation.

To further analyze the performance of the three-group classification model, we focused on the ER group and evaluated its classification against the combined HC and DR groups. Although the original model was designed for multi-class classification (HC, ER, DR), we extracted results specifically for ER vs. non-ER classifications. This analysis enabled the construction of an ROC curve, representing the model's ability to distinguish ER from the other groups (Figure 2). The ROC analysis highlights the discriminative performance of the model, with AUCs indicating that the combined domain achieved the highest predictive accuracy (AUC = 0.93, Figure 2C). This was followed by the conventional domain (AUC = 0.85, Figure 3A) and the VR-based domain (AUC = 0.82, Figure 3B).

### 3.4 Feature importance analysis

The feature importance analysis, based on SHAP values estimated from the CatBoost model, is presented as a bar plot in Figure 3. Out of 67 features, the top 10 predictors included variables from the conventional domain (PDSS, ASI, depression subscale of HADS, and avoidance subscale of LSAS) and the VR-based domain (M2-P-AS, M1-P-SDNN, M2-MR-LF/HF, M1-E6-AS, M1-P-VLF, and M3-HV-LF/HF). This reflects significant contributions from conventional and VR-based domains. The contribution of each domain, variable category within the VR-based domain, and modules of the VR-based domain to the importance values are visualized in Figures 3B–D, respectively. Across domains, the conventional domain demonstrated a higher importance value sum than the VR-based domain (Figure 3B). Within the VR-based domain, Module 2 yielded the highest importance value sum, followed by Module 1, Module 3, and baseline (Figure 3D). Lastly, among the variable categories in the VR-based domain, AS variables exhibited a higher importance value sum than HRV variables (Figure 3C).

## 4 Discussion

This study provides initial evidence supporting the utility of a VR-based assessment in predicting ETR among patients with PD. The model incorporating subjective and physiological measures within immersive VR environments demonstrated superior performance in identifying ER, relative to models based solely on conventional assessment data. In addition, subjective experiences and biosignals collected within VR environments contributed to predictive value comparable to that of conventional clinical measures, offering unique insights that extend beyond traditional assessment tools.

The integration of VR-based and conventional predictors enhanced the model's classification of ER and DR, while maintaining high accuracy in identifying healthy controls. This approach mirrors previous findings where the inclusion of objective physiological data such as HRV and neuroimaging improved prediction in psychiatric treatment response models (15, 41, 50). These results are particularly notable given prior findings on HRV-based prediction. Prior research has shown that HRV parameters were not reliable predictors of pharmacotherapy response in PD (51), and findings have been similarly inconsistent in generalized anxiety disorder (52, 53). One critical advancement in the present study is the use of HRV recorded and subjective anxiety scores during immersive and ecologically valid VR scenarios, rather than relying on resting-state or artificial cognitive stressors. This approach enabled the detection of anxiety-specific physiological patterns that were previously obscured in less dynamic contexts.

Although predictive accuracy is important, one of the key advantages of VR-based assessments lies in their enhanced interpretability. In this study, conventional clinical measures did not differentiate ER from DR, suggesting that these traditional indicators may be insufficient to capture early therapeutic shifts during pharmacotherapy (14). Conversely, the VR-based domain revealed consistent and significant differences between ER and DR groups across multiple scenarios. These included anxiety-inducing, interoceptive, and even post-stimulus relaxation conditions—contexts that evoke subtle emotional and physiological reactions. Importantly, ERs exhibited lower subjective anxiety throughout these immersive scenarios, while appearing indistinguishable from HCs. This highlights the ecological sensitivity of VR-based assessments, which can elicit and measure clinically meaningful variance in real-time emotional reactivity (1). Such granularity not only supports early differentiation between treatment trajectories but also enhances the clinician's ability to interpret behavioral and physiological markers in context, thus offering a more dynamic and individualized understanding of patient change.

The SHAP feature importance analysis revealed that subjective anxiety scores, derived from participant responses during immersive VR experiences, demonstrated unexpectedly high predictive value—comparable to that of physiological HRV metrics. This is particularly noteworthy given that HRV has traditionally been emphasized as an objective and quantitative biomarker in psychiatric research (41). In contrast, subjective anxiety ratings—often collected via VAS—have historically been viewed as less robust due to limitations in discriminative power, standardization, and contextual relevance (54–56). However, the structured and ecologically valid nature of VR environments appears to overcome many of these limitations. Because all participants are exposed to the same controlled anxiety-inducing scenarios, subjective responses become more directly comparable and interpretable (57). These findings highlight that, within VR settings, subjective experience is not merely a supplementary input but a core signal that reflects clinically meaningful emotional reactivity.

This study has some limitations. First, the small sample size, particularly the number of ERs (*n* = 7), constrains statistical power and raises concerns about the stability of machine-learning results. Although LOOCV was used to mitigate overfitting, external replication in larger multi-site cohorts will be necessary to confirm generalizability. Second, participants were not drug-naïve, and medication heterogeneity may have introduced uncontrolled variance. Third, the absence of an independent test dataset, despite use of LOOCV, limits claims regarding model generalizability. Fourth, all participants were recruited from a single center, which may reduce the ecological diversity of the sample. Furthermore, the VR-induced anxiety state may not fully capture the spontaneous and unpredictable nature of panic attacks, which are central to the clinical complexity of PD. Future studies should prioritize replication in larger, multi-site cohorts and consider integration of neuroimaging, cognitive, and ecological momentary data to further elucidate mechanisms underlying ETR. Standardization of VR-based assessment protocols across studies will be essential for establishing normative data and enabling cross-study comparisons.

The encouraging results, obtained even in a small sample, highlight the potential clinical utility of VR-based assessment for predicting ETR in patients with PD. By capturing subjective experiences and physiological signals within standardized, ecologically valid environments, the approach enables early identification of treatment trajectories that are not discernible through conventional assessments alone. The integration of immersive VR technology provides predictive precision and interpretive depth, offering a path toward more personalized, adaptive interventions in clinical psychiatry. Replication in larger and multicenter cohorts is warranted to consolidate these preliminary but promising findings. These findings lay important groundwork for the future development of scalable VR-based tools that can complement traditional evaluations and optimize early decision-making in pharmacological treatment planning.

## Data Availability

The datasets generated and analyzed in this study include sensitive clinical information collected in a hospital setting and are therefore not publicly available. De-identified data may be obtained from the corresponding author upon reasonable request and subject to approval by the Institutional Review Board and institutional data-export committee.
