# Supplementary material for: Simulated virtual reality experiences for predicting early treatment response in panic disorder
Source: Front Digit Health. 2025 Nov 6;7:1684001. doi: 10.3389/fdgth.2025.1684001 (PMC12631630; doi:10.3389/fdgth.2025.1684001)
Supplement: Supplementary file 1 [file Datasheet1.pdf]

Supplementary Material

Simulated Virtual Reality Experiences for Predicting Early Treatment Response in Panic Disorder

Byung-Hoon Kim[1,2], Jae-Jin Kim[1,2], Junhyung Kim[3,4,*], Jiook Cha[5,6,7,8], Sang-Won Jeon[3,4], Kang-Seob Oh[3], Dong-Won Shin[3], Sung Joon Cho[3,4]

*** Correspondence:** Junhyung Kim: jihndy.kim@samsung.com

1 Supplementary Figures

1.1 Supplementary Figure 1

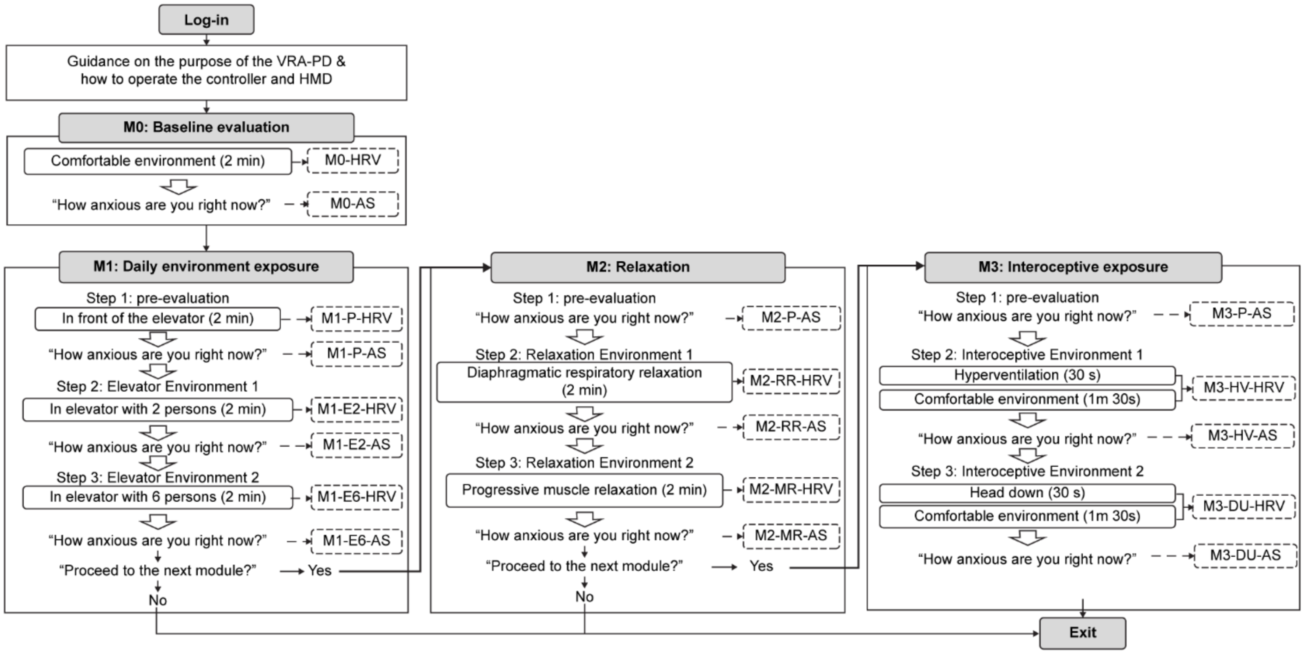

Supplementary Figure 1. Configuration schematic of the Virtual Reality Assessment for Panic Disorder (VRA-PD). A rounded-corner box inside an angled rectangle represents a module within the virtual world. An empty arrow indicates a procedure evaluating whether to proceed within the virtual environment. Items delineated by dotted lines denote documented variables. HMD, head-mounted display; M, module; HRV, heart rate variability; AS, anxiety scores; RR, diaphragmatic respiratory relaxation; MR, progressive muscle relaxation; HV, hyperventilation; DU, head down.

1.2 Description of each module in used in VRA-PD accompanied by screenshots

1.2.1 Virtual Reality Assessment of Panic Disorder (VRA-PD)

1.2.1.1 Purpose of VRA-PD

Virtual reality-based assessments (VRA) for patients with panic disorder (PD) serves as an anxiety behavior evaluation system. The virtual environmental data used in the VRA-PD is based on the modifications of a VR-based relaxation self-training program for PD, with its feasibility confirmed by Jeong et al. [1] and Kim et al. [2]. Both subjective anxiety and physiological responses were achieved in virtual environments with the aim of inducing anxiety-related behaviors in the VRA-PD. The scenario comprises modules that represent different parts of cognitive behavioral therapy for anxiety disorders. These modules included a claustrophobic and social environment (getting on an elevator), interoceptive sensations (hyperventilation (HV) and head down (HD)), and relaxation training.

1.2.1.2 System configuration and operation

Here, the virtual environments used in the VRA-PD were produced through the modification of a pre-existing VR-based relaxation self-training program; additionally, this has been validated as an effective treatment strategy. However, the virtual environment of the "daily environmental exposure module" (module 1) produced via animated graphics was not effective. The remaining three virtual environments were created with a 3D video filmed in a real scene using a 360-degree 3D camera (Insta360 Pro, Insta360 Inc., Irvine, CA).

The virtual environments were exhibited using through a head mounted display (HMD) comprising an Oculus Quest 2 and two Oculus Touch Controllers (Meta, Menlo Park, CA), which produced a 360-degree view with an 89-degree field of vision. Users can independently utilize VRA-PD by following the built-in instructions, which are provided as either text on the screen or audibly through the audio system.

To ensure its validity, Quest 2 and the physiological data acquisition system were connected to the same laptop to interlock the physiological data and VRA-PD. Here, a trained researcher monitored the progress through a laptop screen. User-friendliness was ensured VRA-PD processes by clicking simple icons on the screen with a spear-shaped pointer via a controller. When it was no longer feasible to continue experiencing the virtual environment due to cybersickness or rising anxiety, the user was notified that the evaluation could be terminated by removing the HMD.

Screenshots of VRA-PD

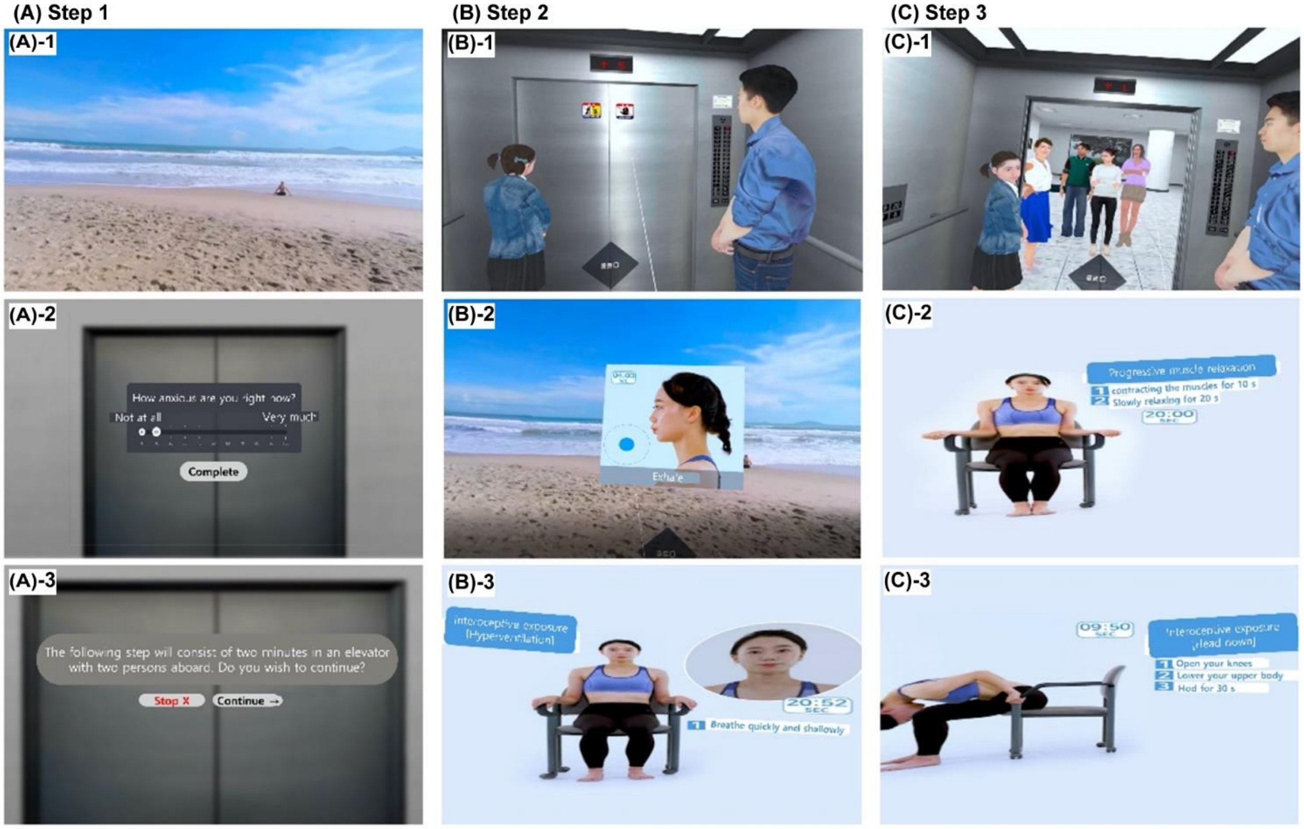

Supplementary Figure 2. Screenshots depicting each module and their scenarios and how the assessment should be conducted.

Contents of each module

Baseline evaluation module (Module 0)

Module 0 was designed to assess the baseline (initial) state of the user before exposure to the virtual environment of the module. Furthermore, the evaluation method used in each module was repeated to familiarize the users with the method. Users were instructed to rest for 2 minutes in a comfortable virtual environment that consisted of a beach scene with a clear sky (Supplementary Figure 2A-1). During this stage, the physiological data of the user in calm state was measured, then the users' subjective anxiety experience was evaluated afterward.

Daily environment exposure module (Module 1)

Module 1 was designed to assess anticipatory anxiety in daily environments and changes in anxiety according to changes in stress intensity. This virtual environment was designed with an elevator boarding environment. Module 1 consisted of three virtual environments: (1) evaluation of anticipatory anxiety by standing in front of an elevator; elevators in which (2) 2 and (3) 6 passengers rode together. When the user selected the "Daily environment exposure" module, the screen was converted into virtual environment (1) (Supplementary Figure 2A-2). In the evaluation of anticipatory anxiety, users were instructed to imagine a scene of the boarding an elevator with 2 and 6 passengers for 2 minutes, respectively (Supplementary Figure 2B-1, 1C-1). Physiological data

acquisition was conducted during each module for 2 minutes. Subjective anxiety score (AS) ratings occurred after each module and were performed in triplicate (Supplementary Figure 2A-3).

Relaxation module (Module 2)

Module 2 was designed to evaluate the relaxation ability of patients with anxiety disorders. This module was designed to experience 2 virtual environments subsequently. These experiences were associated with diaphragmatic breathing relaxation and progressive muscle relaxation. When the user completed Module 1 and pressed the "Next" icon, the screen was converted into a virtual beach environment scene that was used in Module 0. In this module, the user listened to the voice prompt stating the module's objectives and content, and the subjective AS was rated. Notably, physiological data were not acquired in this scene. In a virtual environment related to diaphragmatic breathing relaxation and progressive muscle relaxation, users were instructed to relieve tension by diaphragmatic breathing or forcefully contracting the muscles for 10 seconds and then slowly relaxing them for 20 seconds. Users underwent relaxation exercise that was done as per the instructional video provided by the assistant. This video was displayed at the center of the screen for 2 minutes (Supplementary Figure 2B-2, 2C-2). Physiological data acquisition and subjective AS ratings were performed twice for each virtual environment.

Interoceptive exposure module (Module 3)

Module 3 was designed to assess the users' sensitivity to interoceptive stimuli associated with anxiety disorders. This module precedes Module 2 and users have the option to select it. Once selected, a voice prompt instructs the user in the same manner as it does in Module 2. The user proceeds to undergo a pre-state self-rating anxiety evaluation in the same way as per Module 2. Afterward, users experience 2 virtual environments subsequently, as the video of the assistant's motion played in the center of the screen for HV and HD, respectively (Supplementary Figure 2B-3, 2C-3). Due to the challenges associated with interoceptive stimuli and the risk of inducing panic attacks, both virtual environments performed HV and HD for the first 30 seconds, whereas the remaining 1 minute and 30 seconds was designed to look at a comfortable environment, viz. the beach. Physiological data acquisition and subjective AS ratings were performed twice for each virtual environment.

1.3 Supplementary References

1. Jeong HS, Oh J, Paik M, Kim H, Jang S, Kim BS, et al. Development and feasibility assessment of virtual reality-based relaxation self-training program. Front Virtual Real (2022) 2:72558. doi:10.3389/frvir.2021.722558.

2. Kim B-H, Kim J-J, Oh J, Kim S-H, Han C, Jeong H-G, et al. Feasibility of the virtual reality-based assessments in patients with panic disorder. Front Psychiatry (2023) 14:1084255. doi:10.3389/fpsyt.2023.1084255

1.4 Supplementary tables

Supplementary Table 1. Structure and measurements of the VR-based domain with VRA-PD

Module	Virtual environment	Anxiety scores	HRV parameters
**Baseline evaluation (M0)**	Comfortable beach	M0-AS	M0-HRV*
**Daily environment exposure (M1)**	Pre-evaluation (P)	M1-P-AS	M1-P-HRV*
	In elevator with two persons (E2)	M1-E2-AS	M1-E2-HRV*
	In elevator with six persons (E6)	M1-E6-AS	M1-E6-HRV*
**Relaxation**	Pre-evaluation (P)	M2-P-AS	
	Respiratory relaxation (RR)	M2-RR-AS	M2-RR-HRV*
	Progressive Muscle relaxation (MR)	M2-MR-AS	M2-MR-HRV*
**Interoceptive exposure**	Pre-evaluation (P)	M3-P-AS	
	Hyperventilation (HV)	M3-HV-AS	M3-HV-HRV*
	Head down and up (DU)	M3-DU-AS	M3-DU-HRV*

HRV, heart rate variability; VRA-PD, Virtual Reality Assessment for Panic Disorder.

* Each HRV variable consists of five distinct parameters: low-frequency to high-frequency power ratio (LF/HF), very low-frequency power (VLF), root mean square of successive differences (RMSSD), standard deviation of NN intervals (SDNN), and total power (TP). These five HRV parameters were treated as separate features in the analysis (e.g., M0-LF/HF, M0-VLF, etc.).

Supplementary Table 2. VRA-PD anxiety scores and HRV parameters in among the different groups

Variable	HC (n=27)		ER (n=7)		DR (n=18)		H-value†	post-hoc test‡		
	Mean	SD	Mean	SD	Mean	SD	*p-value*	HC vs ER	HC vs DR	ER vs DR
M0-AS	10.74	20.37	4.29	5.35	33.33	33.95	8.04 (0.018)	1.000	0.029	0.103
M1-P-AS	5.19	12.21	8.57	10.69	32.78	24.69	22.13 (<0.001)	1.000	<0.001	0.059
M1-E2-AS	5.19	12.82	14.29	25.07	34.44	23.57	19.19 (<0.001)	0.672	<0.000	0.200
M1-E6-AS	5.93	12.79	17.14	24.98	46.67	29.31	22.91 (<0.001)	0.663	<0.000	0.106
M2-P-AS	4.81	12.21	5.71	7.87	40.00	27.65	24.25 (<0.001)	1.000	<0.001	0.014
M2-RR-AS	4.07	10.47	4.29	7.87	30.56	27.33	20.50 (<0.001)	1.000	<0.000	0.016
M2-MR-AS	2.22	8.01	2.86	4.88	21.67	23.07	19.20 (<0.001)	1.000	<0.001	0.052
M3-P-AS	4.07	9.31	4.29	7.87	30.56	29.20	19.17 (<0.001)	1.000	<0.000	0.018
M3-HV-AS	5.56	10.86	5.71	7.87	30.00	26.57	15.06 (<0.001)	1.000	<0.000	0.075
M3-DU-AS	3.33	10.00	1.43	3.78	27.22	29.47	17.99 (<0.001)	1.000	<0.001	0.010
M0-SDNN	36.71	12.73	69.41	82.95	34.70	16.04	1.85 (0.396)	1.000	1.000	0.537
M0-RMSSD	30.94	19.11	63.96	99.29	31.60	23.75	0.19 (0.911)	1.000	1.000	1.000
M0-TP	1752.05	3403.86	7769.95	16657.89	1103.69	1094.15	3.54 (0.171)	0.916	0.671	0.212
M0-VLF	452.51	473.75	893.31	1065.03	328.62	276.63	1.41 (0.493)	1.000	1.000	0.834

M0-LF/HF	1.26	1.01	1.34	1.06	1.30	1.35	0.79 (0.675)	1.000	1.000	1.000
M1-P-SDNN	37.40	14.04	65.17	75.33	35.21	19.33	2.56 (0.278)	1.000	0.819	0.391
M1-P-RMSSD	29.57	16.71	65.50	108.63	31.81	25.08	0.32 (0.854)	1.000	1.000	1.000
M1-P-TP	1512.38	2044.72	9042.29	20869.32	1196.50	1337.78	2.70 (0.259)	1.000	0.851	0.345
M1-P-VLF	476.97	577.40	793.84	1026.87	343.94	454.27	4.09 (0.129)	0.768	0.605	0.152
M1-P-LF/HF	1.19	1.20	1.10	0.78	1.53	1.68	0.29 (0.863)	1.000	1.000	1.000
M1-E2-SDNN	42.03	12.59	66.97	82.14	42.17	26.60	1.37 (0.505)	1.000	0.762	1.000
M1-E2-RMSSD	35.26	19.44	75.31	116.49	40.88	39.40	0.78 (0.679)	1.000	1.000	1.000
M1-E2-TP	2344.70	3405.16	7622.55	17416.95	1716.73	3083.16	3.688 (0.158)	1.000	0.171	1.000
M1-E2-VLF	594.19	539.07	1023.96	1682.62	359.02	331.41	3.27 (0.195)	1.000	0.220	1.000
M1-E2-LF/HF	1.31	0.84	1.11	0.49	1.89	2.04	0.10 (0.952)	1.000	1.000	1.000
M1-E6-SDNN	42.66	14.46	58.94	74.71	44.53	32.76	2.72 (0.257)	0.471	0.671	1.000
M1-E6-RMSSD	34.32	24.31	58.87	96.37	45.13	45.48	0.48 (0.789)	1.000	1.000	1.000
M1-E6-TP	1609.50	1099.93	7140.82	16343.37	3819.01	9223.33	2.843 (0.241)	0.511	0.547	1.000
M1-E6-VLF	529.42	426.25	694.26	1251.63	759.92	1579.00	2.17 (0.337)	0.479	1.000	1.000
M1-E6-LF/HF	1.65	1.17	1.75	1.12	1.51	1.81	1.66 (0.436)	1.000	0.648	1.000
M2-RR-SDNN	67.79	18.45	81.70	70.77	55.70	34.97	7.35 (0.025)	1.000	0.020	0.641
M2-RR-RMSSD	52.38	31.86	76.71	112.80	42.91	44.72	4.55 (0.103)	1.000	0.099	1.000
M2-RR-TP	4458.17	5387.15	12925.89	27109.06	2462.22	2095.42	5.44 (0.066)	1.000	0.063	0.632

M2-RR-VLF	864.47	923.28	1640.53	2599.15	411.88	424.18	7.27 (0.026)	1.000	0.048	0.108
M2-RR-LF/HF	9.14	7.80	10.39	10.92	11.36	14.64	0.71 (0.965)	1.000	1.000	1.000
M2-MR-SDNN	60.11	26.46	61.42	20.72	65.89	33.56	0.33 (0.847)	1.000	1.000	1.000
M2-MR-RMSSD	43.63	43.96	41.73	33.27	66.33	54.36	1.612 (0.447)	1.000	0.671	1.000
M2-MR-TP	3875.01	4154.58	2356.84	1563.91	3876.05	3654.28	0.22 (0.896)	1.000	1.000	1.000
M2-MR-VLF	1567.43	1486.25	884.12	1262.61	1264.07	1098.60	2.05 (0.359)	0.458	1.000	0.889
M2-MR-LF/HF	4.64	4.86	3.76	3.12	2.60	3.20	3.87 (0.144)	0.484	0.199	1.000
M3-HV-SDNN	58.71	30.10	56.36	19.54	42.27	26.53	7.15 (0.028)	0.211	0.033	1.000
M3-HV-RMSSD	39.39	51.24	28.79	18.38	28.20	27.83	4.85 (0.088)	1.000	0.083	0.970
M3-HV-TP	2962.11	3933.30	2088.57	789.95	1870.10	2703.42	5.62 (0.060)	1.000	0.078	0.292
M3-HV-VLF	962.85	803.75	1237.07	786.36	891.89	932.72	2.59 (0.275)	0.936	1.000	0.341
M3-HV-LF/HF	4.31	4.48	3.38	3.17	3.14	2.90	0.77 (0.682)	1.000	1.000	1.000
M3-DU-SDNN	64.84	29.05	45.21	14.18	58.79	33.69	4.02 (0.134)	0.150	0.999	0.685
M3-DU-RMSSD	51.62	48.24	30.40	16.04	53.48	48.19	0.88 (0.644)	1.000	1.000	1.000
M3-DU-TP	3325.57	5500.96	1414.27	919.48	2549.72	4443.07	3.81 (0.149)	0.637	0.218	1.000
M3-DU-VLF	1004.04	857.17	432.56	314.60	715.06	807.31	4.88 (0.087)	0.154	0.360	1.000
M3-DU-LF/HF	2.23	2.36	1.74	1.98	1.58	1.38	2.49 (0.288)	0.752	0.532	1.000

VRA-PD, Virtual Reality Assessment of Panic Disorder; HC, healthy control; ER, early response; DR, delayed response; SD, standard deviation; HRV, heart rate variability; AS, anxiety score; RR,

diaphragmatic respiratory relaxation; MR, progressive muscle relaxation; HV, hyperventilation; DU, head down; LF, low frequency; HF, high frequency; VLF, very low frequency; TP, total power; SDNN, standard deviation of NN intervals; RMSSD, root mean square of successive differences.

† Kruskal–Wallis H test was used instead of analysis of variance due to small sample size in the ER group (n=7).

‡ Post-hoc comparisons were performed using Mann–Whitney U tests with Bonferroni correction for multiple comparisons.

$p < 0.05$, statistically significant.
